# Accurately Shaping Supercontinuum Spectrum via Cascaded PCF

**DOI:** 10.3390/s20092478

**Published:** 2020-04-27

**Authors:** Jifang Rong, Hua Yang, Yuzhe Xiao

**Affiliations:** 1 College of Computer Science and Electronic Engineering, Key Laboratory for Micro/Nano Optoelectronic Devices of Ministry of Education, Hunan University, Changsha 410082, China; rongjifang@hnu.edu.cn; 2 Synergetic Innovation Center for Quantum Effects and Application, Hunan Normal University, Changsha 410082, China; 3 Department of Electrical Engineering, University of Wisconsin-Madison, Madison, WI 53715, USA; xiao5@wisc.edu

**Keywords:** spectral shaping, photonic crystal fiber cascade, zero-dispersion frequencies spacing, supercontinuum generation

## Abstract

Shaping is very necessary in order to obtain a wide and flat supercontinuum (SC). Via numerical simulations, we accurately demonstrated shaping the SC using the fiber cascading method to significantly increase the width as well as the flatness of the spectrum in silica photonic crystal fiber (PCF). The cascaded PCF contains two segments, each of which has dual zero-dispersion frequencies (ZDFs). The spectral range of the SC can be expanded tremendously by tuning the spacing between the two ZDFs of the first segmented cascaded PCF. Increasing the pump power generates more solitons at the red edge, which accelerates solitons trapping and improves the spectral flatness of the blue edge. Furthermore, cascading the second segmented PCF by choosing appropriate fiber parameters ensures the flatness of the red end of SC. Therefore, a cost-effective alternative method for broad and flat supercontinuum generation in the near-infrared range is proposed here, which can be implemented easily in any photonics laboratory, where dual ZDFs PCFs are commonly found.

## 1. Introduction

Since Alfano and Shapiro firstly reported supercontinuum (SC) in 1970 in bulk glass [1], it has been explosively used in various fields, including spectroscopy [2], optical coherent tomography [3], metrology [4], and so on. Up to now, both numerical simulations and experiments have shown that SC can be obtained by the extremely nonlinear broadening of narrow-band incident pulses, whose widths vary from femtosecond to picosecond [5]. As is well known, the distinguishing features of SC are related to spectral range and spectral flatness, both of which are essential to promoting the technical applications of SC. The femtosecond pulse pumped in the anomalous dispersion region can lead to abundant spectral components and relatively broadband spectrum, via soliton dynamics [5,6,7]. Therefore, most of the studies on SC have chosen femtosecond pulses as the pump sources. In this case, stimulated Raman scattering (SRS) and high-order dispersion (HOD) are crucial to the evolution of ideally periodic high-order soliton [8,9,10,11]. Accordingly, the high-order soliton splits and sheds away energy to resonant nonsolitonic radiation, depending on the phase-matching condition [5]. When the group velocity matches between solitons and dispersion waves (DWs), stable trapping dispersion waves (Trapping DWs) can be generated under the effect of four-wave mixing (FWM) [12]. The contribution of Raman solitons, DWs, and Trapping DWs to SC has been investigated thoroughly in photonic crystal fiber (PCF) [13,14,15].

Recently, studies on ultrafast dynamics in mode-locked lasers have broadened our horizons in complex nonlinear systems and contributed to laser design. It is convenient for us to obtain a femtosecond pulse with few-cycle and high peak power as a pump source for supercontinuum generation (SCG). That further revives interests in broad and flat SCG [16,17]. Numerous studies have shown that red-shifted dispersion wave (R-DW) and blue-shifted dispersion wave (B-DW) can be generated simultaneously in photonic crystal fibers (PCFs) with two or more zero-dispersion frequencies (ZDFs) to improve spectral width. For example, the authors of [18,19,20,21,22,23,24,25] show that the DWs and Trapping DWs can contribute to the generation of wider or smoother SC. Poletti et al. have mathematically explained the soliton spectral tunneling effect in an index-guiding holey fiber with an adjustable group velocity dispersion (GVD) barrier over a large frequency range, which can be used to adjust the spectral width [26]. All these results show that increasing the number of ZDFs will increase spectral width, while spectral flatness will decrease. Subsequently, many works have shown that the cascaded method can improve the spectral flatness or spectral width of SC [27,28,29]. For example, Guo et al. have shown that soliton spectral tunneling effect in a segmented cascaded PCF with engineered dispersion can prompt the soliton pulse to travel over a wide spectrum range [27]. Chunyu Guo et al. have experimentally demonstrated a flat SCG in cascaded fibers pumped by a continuous wave laser, but the spectrum width is just 420 nm at −10 dB level [28]. Saili Zhao et al. have reported harnessing rogue waves in cascaded PCFs for SCG [29]. Haihuan Chen et al. have reported the fabrication of cascaded PCF tapers in monolithic design, and demonstrated flat broadband SCG in cascaded PCF tapers [30]. All of these cascaded methods have improved spectral span or spectral flatness to some extent, but few works have acquired large bandwidth and high flatness simultaneously. As a result, much research about SCG has focused on chalcogenide PCFs whose bandwidth can reach microscale and flatness is fine too, due to its wider transmission window into long-wavelength range and higher nonlinearity [31,32,33,34]. However, the poor thermal stability of this material is not conducive to the widespread industrial application of SC, not replacing the position of silica PCF in SCG. More recently, the research on SCG has grown more and more extensive, even extending to the generation of SCG by dark solitons [35,36,37]. Therefore, it is meaningful to accurately shape the SC to obtain a wide and flat supercontinuum spectrum in silica PCF.

In this paper, we propose a method of accurately shaping SC via cascaded PCF. Here, each PCF needs to be tapered and spliced to the next stage; the splicing loss is directly ignored to get a better physical understanding of various effects. In the first part of our simulation, several PCFs with different ZDF spacing are used as the propagation medium of the first segmented cascaded PCF. We found that increasing the ZDF spacing is beneficial for extending the SC by R-DW and trapping blue-shifted dispersion wave (Trapping B-DW). Although increasing pump power is adequate to generate more solitons, accelerating solitons capture will improve the spectral flatness of the blue edge. Then, in the second part of our simulation, we analyze the output spectrum of the first segment and appropriately choose the fiber parameters of the second segmented cascaded PCF, realizing the accurate shaping of the red edge spectrum.

## 2. Propagation Model

Femtosecond pulse propagation in cascaded single-mode PCF can be described by the generalized nonlinear Schrödinger equation(G-NLSE) [5,18]:

(1) $$\begin{array}{cc} \frac{\partial A(z,T)}{\partial z} & =\sum_{k\geq 2}\frac{i^{k+1}\beta_{k}}{k!}\frac{\partial^{k}A}{\partial T^{k}}\\ & +i\gamma \left(1+\frac{i}{\omega_{0}}\frac{\partial }{\partial T}\right)\times ([A\left(z,T\right)\int_{-\infty }^{+\infty }R\left(T^{\prime }\right)|A\left(z,T-T^{\prime }\right){|}^{2}dT^{\prime }+i\Gamma_{R}\left(z,T\right)]), \end{array}$$

where is A(z,T) complex amplitude of time-domain pulse envelope; *T* and *z* are, respectively, the time parameter related to group velocity at the central frequency $\omega_{0}$ and propagation distance of optical pulse; γ is the nonlinear coefficient of the optical fiber; $\beta_{k}$ is the kth-order dispersion coefficient at the central frequency $\omega_{0}$; $\Gamma_{R}$ describes thermally-driven spontaneous Raman scattering [5]; and R(T) is the nonlinear response function:

(2) $$R\left(T\right)=\left(1-f_{R}\right)\delta \left(T\right)+f_{R}h_{R}\left(T\right),$$

which includes Raman contribution and Kerr components. We use $f_{R}$ = 0.18 and $h_{R}\left(T\right)$ determined from the fused silica Raman cross section [5,10]. Dispersion effects relate to the first term on the right side hand of Equation (1) as well as nonlinear optical effects correspond to the second one including self-phase modulation (SPM), SRS, and self-steepening (SS). The linear loss of fiber is neglected because only a short transmission distance is considered. We adopted the non-chirped Gaussian pump pulse propagating in cascaded PCF: $A\left(0,T\right)=\sqrt{P_{0}}\exp\left(T^{2}/2T_{0}^{2}\right)$.

## 3. Numerical Simulation and Discussion

**Figure 1:**
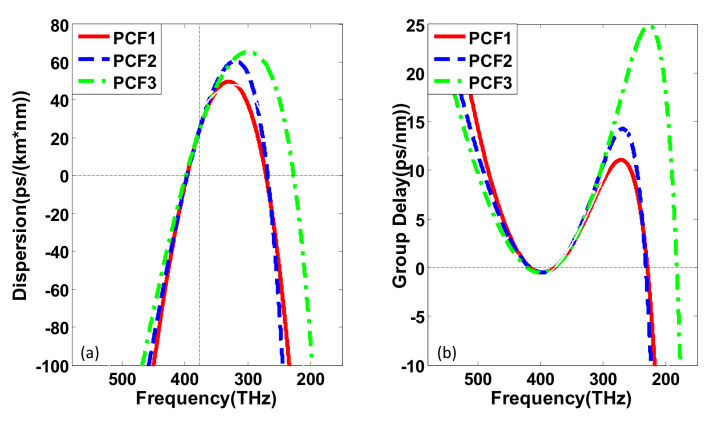
(**a**) The dispersion curve and (**b**) relative group delay curve of the adopted PCFs. Among them, red solid line, blue dashed line, and green dash-dot line represent PCF1, PCF2, and PCF3 respectively. The vertical dashed line in panel (**a**) marks the pump wavelength λ = 795 nm (375 THz).

We adopt the split-step Fourier method with adaptive step size control to solve the G-NLSE [10,38,39,40,41]. Based on the G-NLSE, we will discuss the evolution process of the femtosecond pulse pumped in the anomalous dispersion region of the cascaded PCF. In the first section, PCFs, specifically PCF1, PCF2, and PCF3, which can be obtained by properly setting the geometry of the air–silica holey fiber according to the method proposed in [42,43], are candidates for the first segmented cascaded PCF to demonstrate how the spacing of ZDFs influences the output spectrum. The dispersion profile and relative group delay of PCF1, PCF2, and PCF3 as a function of the frequency are depicted in Figure 1a,b and the dispersion parameters of them are shown in Table 1. In Figure 1a, we can see that the two ZDFs divide the whole dispersion profile into three regions: the left and right regions are normal dispersion regions, and the middle part is the anomalous dispersion region. Meanwhile, the first (high-frequency) and the second (low-frequency) ZDFs provide positive and negative dispersion slopes, respectively. Here, $d_{PCF1}$ = 125 THz, $d_{PCF2}$ = 127 THz, and $d_{PCF3}$ = 178 THz represent the frequency spacing between the two ZDFs of PCF1, PCF2, and PCF3, respectively. The parameters of input pulse are chosen as follows; peak power is $P_{0}$ = 1660 W, initial pulse width is $T_{0}$ = 50 fs, which has been proved to be an appropriate temporal width to maintain the coherence of the input pulse [5]. Pump wavelengths are all at λ = 795 nm (375 THz). Assuming that the effective mode areas of the three different fibers are the same, the nonlinear coefficient of the PCFs is γ=0.124 W^−1^m^−1^. It can be seen from Figure 1a that second-order dispersion parameters at pump central frequency of the three PCFs are equal ($\beta_{2}$ = −0.80352 ps${}^{2}$/km) to ensure the same solitons order *N* under the same incident conditions. The soliton order *N* of the input pulse is determined by both pulse and fiber parameters through $N^{2}=L_{D}/L_{NL}$. Here, $L_{D}=T_{0}^{2}/\left|\beta_{2}\right|$ and $L_{NL}=1/\gamma P_{0}$ are the characteristic dispersive and nonlinear length, respectively.

### 3.1. Pulse Evolution in PCFs with Different ZDF Spacing

**Figure 2:**
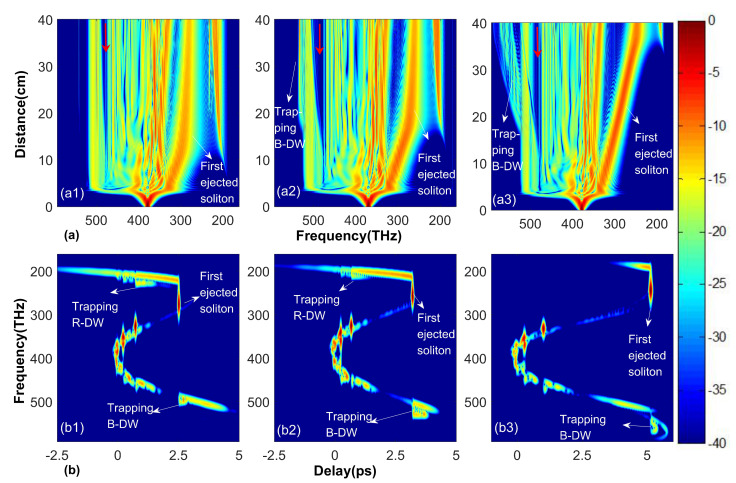
Evolution of the spectral characteristic of the incident optical pulse as a function of fiber length in three PCFs with different zero-dispersion frequency (ZDF) spacing and the corresponding spectrograms at 40 cm. Panels (**a1**,**b1**) correspond to $d_{PCF1}$ = 125 THz, Panels (**a2**,**b2**) correspond to $d_{PCF2}$ = 127 THz, Panels (**a3,b3**) correspond to $d_{PCF3}$ = 178 THz. Here, $d_{PCF1}$, $d_{PCF2}$ and $d_{PCF3}$ stand for the frequency spacing between two ZDFs of PCF1, PCF2, and PCF3, respectively. Red arrows in Panels (**a1**–**a3**) represent spectrum gaps in B-DW.

To deeply discuss the influence of ZDF spacing in the first segmented cascaded PCF on SC, in Figure 2, we compare the evolution process of pulse spectrum with different ZDF spacing in 40 cm PCFs as well as the corresponding numerical cross-correlation frequency-resolved optical gating (X-FROG) trace at the output of PCFs. As shown in Figure 2a, during the initial stage of pulse propagation, the symmetrical spectrum broadening by SPM is observed. Subsequently, the higher-order soliton splits into fundamental solitons with different peak powers and almost simultaneously transfers energy to phase-matched B-DW. After soliton fission, the ejected fundamental solitons continuously red-shift to the low-frequency region under the effect of Raman induced soliton self-frequency shifting (SSFS). The first ejected soliton has a higher intensity and faster red-shifted speed than other solitons and experiences greater frequency downshift relative to the central frequency. This can be verified by spectrum evolution in Figure 2a. Besides, there is a distinct phenomenon by comparing Figure 2(a1–a3), with the increasing of ZDF spacing, the Trapping B-DW has much richer spectral components to broaden the spectrum. The ZDF spacing of PCF3 is larger than that of PCF1 and PCF2, so the first ejected red-shifted soliton in PCF3 has a larger red-shift distance to arrive at the second ZDF. From Figure 1b, we know that the time delay increases and the group velocity decreases with soliton red-shift. Therefore, the first ejected red-shifted soliton can gradually mix with B-DW from the low-frequency to the high-frequency in PCF3, leading to form stable Trapping B-DW at the trailing edge of B-DW. This process completely broadens the spectrum of the blue edge, as can be seen in Figure 2(b3). In contrast with that in PCF3, in PCF1, the Trapping B-DW generated by the interaction between the low-frequency part of B-DW and the first ejected soliton superposes with B-DW in frequency and does not broaden the spectrum, as is shown in Figure 2(b1), while the Trapping B-DW of PCF2 in Figure 2(b2) just broadens the spectrum a little. Besides, in all these three cases, an obvious gap (marked by red arrow) appears in the spectrum region of the B-DW as a result of the FWM effect transferring energy from B-DW to the Trapping B-DW, as shown in Figure 2a. However, trapping red-shifted dispersion waves (Trapping R-DWs) has no obvious effect to SC spectrum broadening. In our simulations, we find that when the input condition remains unchanged, that is, when the soliton order *N* = 8, Trapping B-DW can broaden the spectrum only if $d_{PCF1}$ > 125 THz is satisfied. Therefore, we can conclude that the Trapping B-DW can broaden the spectrum only when the ZDF spacing reaches a certain degree.

Until the central frequency of the first ejected soliton approaches to the second ZDF of PCFs with a negative dispersion slope, Trapping B-DW no longer keeps broadening the spectrum in the blue region, as shown in Figure 2(a2,a3). With the increasing of ZDF spacing, red-shifted soliton that greatly extends the red edge propagates over a wider spectral range to reach the second ZDF where R-DW radiates out according to the phase-matching condition. Thus, the larger the ZDF spacing is, the greater the translation distance and frequency downshift are needed to radiate R-DW, which is consistent with the results shown in Figure 2a. Therefore, the output spectral width of SC is strongly influenced by the ZDF spacing, making it possible to manipulate the SCG process by adjusting the ZDF spacing.

**Figure 3:**
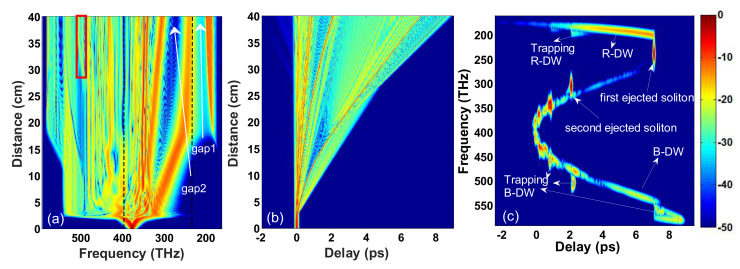
The evolution of the (**a**) spectral and (**b**) temporal signal as a function of the fiber length, and (**c**) the spectrogram at the output of PCF3 when the pump power is increased up to 2600 W. The black vertical dashed lines in panel (**a**) mark the two ZDFs of PCF3. The red rectangle area represents the place Trapping B-DW well fills the spectrum gap in B-DW.

From the above analysis, we find that a large ZDF spacing in PCFs can improve the width of the output spectrum by both R-DW and Trapping B-DW. This, however, leads to a poor flatness of the SC spectrum. The authors of [18] indicate that increasing the pump power is adequate to generate more solitons, which can accelerate soliton trapping to improve spectral flatness. For this purpose, Figure 3 shows the evolution of the optical pulse in PCF3 when the pump power is increased to 2600 W. An important phenomenon observed in Figure 3c is that the second ejected red-shifted soliton overlaps with B-DW and R-DW in the time domain to generate Trapping B-DW and Trapping R-DW by FWM effects. Subsequently, the four pulses co-propagate in the PCF and the generated Trapping B-DW well fills the spectrum gap in B-DW (see the red rectangle region in Figure 3a). That is to say, by reasonable selection of the spacing between ZDFs, a pump power not only can broaden the width of the SC but also can ensure the flatness of the blue edge.

### 3.2. Pulse Evolution in Cascaded PCF

**Figure 4:**
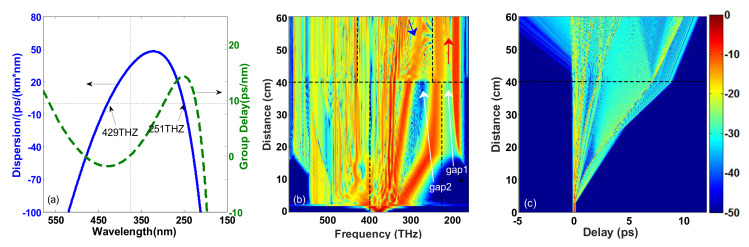
(**a**) The dispersion curve and relative group delay curve of PCF4. Evolution of the (**b**) spectral and (**c**) temporal signal of the input pulse as a function of propagation distance. The black vertical dashed lines in panels (**a**,**b**) mark the pump position of 375 THz and the two ZDFs of the first and second segmented cascaded PCF, respectively. The black horizontal dashed lines in panels (**b**,**c**) mark where PCF3 and PCF4 are connected. The red arrow and the blue arrow point to two filled spectrum gaps respectively.

From the output spectrum of PCF3 in Figure 3, we find that the flatness of the SC is better in the blue region than the red region where the energy is mainly distributed in a few solitons and DWs. There are two reasons for this: First, the Trapping R-DW superimposes with R-DW in the low-frequency region so that the spectrum gap (named gap1, see gap1 in Figure 3a) between R-DW and the first ejected soliton is not filled. Second, the large group velocity difference between the two fundamental solitons leads to a large spectrum gap (named gap2, see gap2 in Figure 3a) between them. The cascaded scheme is given as follows. When the pump pulse is transferred from the first segmented fiber (PCF3) into the second segmented fiber, the second ejected fundamental soliton in PCF3 turns into a higher-order soliton in the second segmented fiber. Then, the higher-order soliton will be split into fundamental soliton, so the red-shifted fundamental soliton and the corresponding R-DW fill the spectrum gap2 and the spectrum gap1, respectively. According to the definition of soliton order $N^{2}=\gamma P_{0}T_{0}^{2}/\left|\beta_{2}\right|$, in order to realize the change from fundamental soliton to higher-order soliton of the second ejected soliton in the second segmented PCF (specifically, a larger value of *N*). The GVD at central frequency $\omega_{0}$ should be less than that of PCF3, or the nonlinear coefficient should be greater than that of PCF3 in the second segmented cascaded PCF. Moreover, the second ZDF of the second segmented fiber needs to be located in the spectrum range of the first ejected soliton at the output end of PCF3, so that the first ejected soliton cannot maintain the soliton shape and is dispersed, which further smoothens the SC spectrum. Based on the above requirements, we choose the appropriate PCF4, whose group delay and group dispersion curves are shown in Figure 4a and the dispersion parameter at the center frequency $\omega_{0}$ of the pump is $\beta_{2}=-11.2346\;ps^{2}$/km, $\beta_{3}=-0.0348\;ps^{3}$/km, $\beta_{4}=2.7608\times 10^{-6}\;ps^{4}$/km, $\beta_{5}=6.9342\times 10^{-8}\;ps^{5}$/km, $\beta_{6}=5.4154\times 10^{-10}\;ps^{6}$/km, $\beta_{7}=-4.2448\times 10^{-12}\;ps^{7}$/km, $\beta_{8}=2.3240\times 10^{-14}\;ps^{8}$/km, $\beta_{9}=-8.3230\times 10^{-17}\;ps^{9}$/km, $\beta_{10}=1.9096\times 10^{-19}\;ps^{10}$/km, $\beta_{11}=-2.5893\times 10^{-22}\;ps^{11}$/km, $\beta_{12}=1.5974\times 10^{-25}\;ps^{12}$/km. The two ZDFs are 429 THz (699 nm) and 251 THz (1195 nm), respectively, and the nonlinear coefficient is γ = 0.193 W${}^{-1}$m${}^{-1}$.

The total length of the cascaded PCF constructed by PCF3 and PCF4 is 60 cm, in which the first 40 cm is PCF3 and the remaining 20 cm is PCF4. The frequency domain and temporal domain evolution of the pump pulse in this cascaded PCF are shown in Figure 4b,c. Here, the pumping conditions are consistent with those in PCF3 in Figure 3. Thus, the evolution of the first 40 cm is the same as that in Figure 3. The changes in dispersion and relative group delay are presented as a bend of the solitons in the temporal domain at a distance of 40 cm, which is shown in Figure 4c, different from Figure 3b. In the frequency domain, only a small part of the energy of the first ejected soliton in PCF3 is still in the anomalous dispersion region. In contrast, most of the energy is in the normal dispersion region the balance between GVD and SPM is broken in PCF4. The spectral broadening of the partial energy in the anomalous dispersion region is much wider than it is in the normal dispersion region. This is due to the chirp generated by the SPM in the anomalous dispersion region, which causes the pulse to compress in the time domain and broaden in the spectral domain. In the normal dispersion region, the process is just the opposite: the partial energy just slightly broadens in the frequency domain due to SPM and diverges rapidly in the temporal domain due to different GVD. In a word, this process enriches the spectrum components which improves the flatness of the red region to a certain extent.

However, the second ejected soliton in PCF3 plays a major role in shaping the SC, which turns into a higher-order soliton, but its central frequency is far from the two ZDFs of PCF4. Therefore, only SRS acts as the perturbation on its transmission and the HOD effect on the spectrum is negligible. Accordingly, only one fundamental soliton (marked by the blue arrow in Figure 4b) is split by the secondly ejected soliton, owing to its weak energy, as shown in Figure 4b. Under the effect of cascaded SRS, SSFS in the subsequent propagation process efficiently fills the spectrum gap2. As the SSFS approaches the second ZDF of PCF4, phase-matched R-DW (marked by the red arrow in Figure 4b) is gradually scattered out, filling gap1. With the increase in propagation distance, R-DW keeps tilting towards the higher frequency and its energy increases continuously in PCF4. When the spectrum recoil effect is balanced with SSFS, SSFS disappears. From the spectrum in Figure 4b, we can clearly see the shaping effect in the low-frequency region.

**Figure 5:**
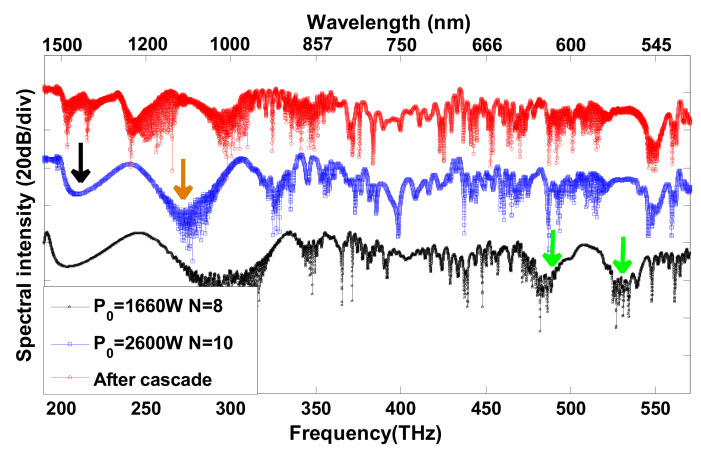
The spectral intensity of three different cases. The black pentagram-symbol line, blue square-symbol line, and red circle-symbol line represent the output spectrum shown in Figure 2(a3), Figure 3a, and Figure 4b, representing without increasing the pump power ($P_{0}$ = 1660 W, *N* = 8), increasing the pump power ($P_{0}$ = 2600 W, *N* = 10), and after cascade ($P_{0}$ = 2600 W, *N* = 10), respectively. The three curves are staggered on the vertical axis only for comparison rather than actual intensity.

In Figure 5, from bottom to top, the three curves corresponding to the output spectrum in Figure 2(a3), Figure 3a, and Figure 4b, represent without increasing the pump power ($P_{0}$ = 1660 W, *N* = 8), increasing the pump power ($P_{0}$ = 2600 W, *N* = 10), and after cascade ($P_{0}$ = 2600 W, *N* = 10), respectively. We use the standard deviation $\sigma =\sqrt{\frac{1}{N}\sum_{n=1}^{N}{\left(x_{i}-\mu \right)}^{2}}$ to describe the flatness of the output spectrum, where $x_{i}$ is the logarithmic representation of the output power at each frequency sampling point, μ is the average of $x_{i}$. The calculated standard deviations of the three mentioned cases above are 11.5, 9.8, and 7.6, respectively, which shows that the flatness from bottom to top is becoming increasingly optimal. This result is consistent with the conclusion in Figure 5. Compared with the black pentagram-symbol line, the flatness of the high-frequency part of the blue square-symbol line improves considerably; the two spectrum gaps on the black pentagram-symbol line indicated by the two green arrows are filled by the Trapping DWs after increasing the pump power. Relative to the blue square-symbol line, there is further improvement in the flatness of the low-frequency part of the red circle-symbol line. The un-flat areas indicated by the orange and the black arrow on the blue square-symbol line are improved by the red-shifted fundamental soliton and the corresponding R-DW in the second segmented cascaded fiber, respectively.

**Figure 6:**
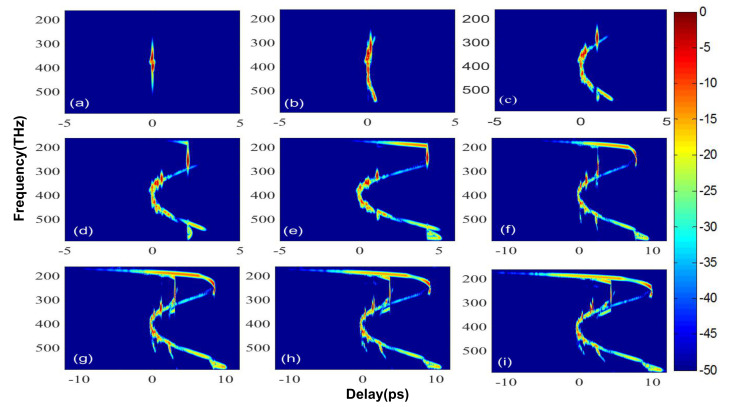
The spectrograms at different propagation distances *z*: (**a**) *z* = 2 cm, (**b**) *z* = 5 cm, (**c**) *z* = 12 cm, (**d**) *z* = 18 cm, (**e**) *z* = 28 cm, (**f**) *z* = 45 cm, (**g**) *z* = 50 cm, (**h**) *z* = 55 cm, (**i**) *z* = 60 cm.

In order to clearly demonstrate the evolution of the pump pulse in the cascaded PCF, we used the X-FROG trace to record the time-frequency diagram of the pulse in nine selected locations. For a short propagation distance of 2 cm, the pump pulse rapidly symmetrical stretches in frequency by SPM (Figure 6a). Propagating from 2 to 5 cm, the pump pulse breaks due to the effects of HOD and SRS, transferring energy to B-DW in the blue region, as shown in Figure 6b. Until reaching 12 cm, the bandwidth of B-DW remains unchanged and its tail starts to tilt back because the high-frequency components of B-DW propagate slower than the low-frequency components in the normal dispersion region. The head edge of B-DW overlaps with the first ejected red-shifted soliton, as shown in Figure 6c. As the distance increases, more high-frequency components of B-DW interact with the slowing down fundamental soliton (see Figure 6d). It is apparent that the Trapping DW moves continuously to the backward of B-DW to maximize the spectrum of the blue region at 18 cm, where the soliton closes to the second ZDF and begins to emit R-DW. When propagating to 28 cm, the second fundamental soliton in front of the first ejected high-intensity soliton effectively mixes with B-DW, which generates the Trapping B-DW and fills in the spectral gap in B-DW frequency region to flatten the blue edge of SC, as seen in Figure 6e. When the pulse propagates for 45 cm in PCF4 (see Figure 6f), the first ejected soliton in PCF3 is dispersed and curved in shape as a result of the change in GVD. Meanwhile, the second ejected soliton in PCF3 splits into a red-shifted fundamental soliton and emits an extra-low energy R-DW simultaneously, which is generated due to the poorly met phase-matching condition. Between 45 and 50 cm, the newly generated fundamental soliton moves close to the second ZDF of PCF4, then R-DW is amplified (Figure 6g). In the last two figures, Figure 6h,i, the frequency shift of the fundamental soliton stops as the energy of the R-DW keeps increasing. The inclination of DWs increases as the pulse propagates further and the flatness of the red region is improved.

## 4. Statistics with Spectral Flatness

**Figure 7:**
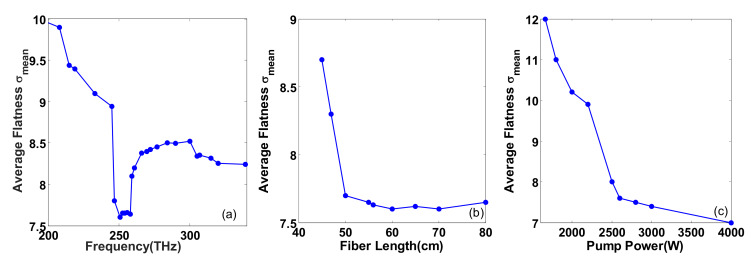
The evolution of the average flatness of 100 individual simulations ($\sigma_{mean}$) as a function of (**a**) the second ZDF in the second segmented PCF, (**b**) fiber length, and (**c**) pump power.

This section mainly discusses the influence of several main factors on the output spectrum flatness of cascaded fiber. In Figure 7, we show average flatness of 100 individual simulations when changing the position of the second ZDF in the second segmented PCF, fiber length, and pump power, and the calculated results are, respectively, demonstrated in Figure 7a–c. The average flatness is defined as $\sigma_{mean}=\frac{\sum_{n=1}^{N}\sigma_{i}}{N}$, where $\sigma_{i}$ is the standard deviation of single simulation. The lower the value of $\sigma_{mean}$, the better the flatness.

From Figure 4b, we can learn that the position of the second ZDF in the second segmented PCF is crucial to the behavior of spectral flatness. When the second ZDF is around 251 THz, the evolution processes of the spectrums are similar to that in Figure 4b. In this case, the newly generated red-shifted soliton in the second segmented fiber and the emitted DWs well fill the spectrum gaps. Therefore, it has a good flatness, which is named as flat area representing a low value of $\sigma_{mean}$ (≈7.6). When the second ZDF moves from the flat area to the lower frequency region, the flatness deteriorates obviously and the value of $\sigma_{mean}$ quickly becomes higher, which arises from the ZDF in lower frequency region leading to a continued red-shift of the two fundamental solitons in the anomalous dispersion region so that the spectrum gaps are almost not filled. On the contrary, when the ZDF moves from the flat area toward the higher frequency region, the flatness slowly declines as the red-shift frequency range of the newly generated soliton is limited and the emitted DW partially overlaps the spectral components of the first ejected soliton. Therefore, spectrum gaps cannot be filled well and the value of $\sigma_{mean}$ shows a slow change. When the second ZDF reaches above 315 THz, where the two fundamental solitons generated in the first segmented fiber are directly translated into DWs in the normal dispersion region, the SC gets smoother and the flatness of the output spectrum is improved to some extent. With the continuous increase of the second ZDF, the average flatness will not change greatly, that is, $\sigma_{mean}$ will stay at a stable value (≈8.2). In a word, the output spectral flatness can be adjusted by the sencond ZDF in the second segmented cascaded PCF.

In Figure 7b, the length of the first segmented fiber is fixed to 40 cm, and when the total length of the fiber is less than 60 cm, the value of $\sigma_{mean}$ decreases with the increase in the fiber length. This is because the red-shift of the newly generated soliton in the second segmented fiber and the generation of DW are limited by the transmission distance. Until the fiber length reaches 60 cm, the best flatness of the spectrum is achieved where the spectrum recoil effect is balanced with SSFS. Continuing to increase the length of the fiber, the flatness of the fiber will not change obviously. As fiber length increases in actual applications, the loss will also increase. In this paper, 60 cm is selected as the optimal length.

In Figure 7c, increasing the pump power generates more solitons at the red edge, which accelerates solitons trapping and improves the spectral flatness, so the value of $\sigma_{mean}$ decreases gradually. When the pump power is less than 2600 W, the flatness is relatively poor because the spectral gap in B-DW is not filled by Trapping B-DW, which is similar to the situation in Figure 2(a3). Until the pump power is increased to 2600 W, the generated Trapping B-DW well fills the spectrum gap in B-DW, as shown in Figure 3a. When the pump power continues to increase, the flatness will gradually improve, but the improved speed is much lower than below 2600 W. Therefore, it is inefficient to continuously increase the pump power to more than 2600 W in order to improve the spectral flatness. This manuscript chooses exactly 2600 W as the optimum pump power.

We can conclude that, specifically in this manuscript, the optimum conditions for large spectral width and high spectral flatness (~1 um at −15 dB) are as follows; pumped at the frequency of 375 THz in the anomalous dispersion region in 60 cm fiber, incident power of 2600 W, the ZDF spacing of the first segmented fiber is 178 THz, and the second ZDF of the second segmented fiber is 251 THz. However, there are potential challenges and limitations in the practical applications of this method, where we need to consider access loss, transmission loss, and splicing loss. It is necessary to increase the pump power appropriately to compensate for actual total loss to ensure the same results as the theory analysis. Besides, when designing ZDF spacing, we need to consider the power endurability of the PCFs to ensure the spectral width and spectral flatness. What is more, it is difficult to achieve accurate control of the dispersion for practical devices, so detailed error analysis mechanism is very important in practical application.

## 5. Conclusions

In this work, we numerically demonstrate accurately shaping the SC to acquire wider and flatter SC via cascaded PCF, which consists of two silica PCFs with dual ZDFs. These results suggest that choosing a larger spacing between the two ZDFs of the first segmented cascaded PCF can not only redistribute energy between DWs and Trapping DWs, but also raise the red-shift of R-DW, which is beneficial to broaden the spectrum. Moreover, increasing the pump power can tremendously increase soliton trapping to improve the flatness of the blue edge of the spectrum. More importantly, we analyzed the output of the first segmented fiber and reasonably selected the second segmented PCF parameters, especially the second ZDF position, to reshape the SC to attain a flatter SC over the entire spectrum. We believe the result of this work can serve as a guideline in SCG, which can promote the technical applications of SC. In a next step, we will study the device with more cascaded PCFs to improve spectral width and spectral flatness more efficiently, which would be an interesting and important subject.

## Figures and Tables

**Table 1:** The dispersion parameters of PCF1, PCF2, and PCF3.

$\beta_{k}\left({\mathrm{ps}}^{k}\right)$/km	PCF1	PCF2	PCF3
$\beta_{2}$	−8.0352	−8.0352	−8.0352
$\beta_{3}$	$6.2527\times 10^{-2}$	0.06662	0.060473
$\beta_{4}$	$6.5314\times 10^{-5}$	$0.9517\times 10^{-5}$	$-7.7942\times 10^{-6}$
$\beta_{5}$	$-3.3086\times 10^{-7}$	$-2.2194\times 10^{-7}$	$-1.6566\times 10^{-7}$
$\beta_{6}$	$1.2379\times 10^{-9}$	$7.7245\times 10^{-10}$	$1.9090\times 10^{-10}$
$\beta_{7}$	$-4.3681\times 10^{-12}$	$-1.0368\times 10^{-11}$	$2.1444\times 10^{-12}$
$\beta_{8}$	$1.7687\times 10^{-14}$	$9.6356\times 10^{-14}$	$1.0424\times 10^{-14}$
$\beta_{9}$	$-8.9723\times 10^{-17}$	$-4.3407\times 10^{-16}$	$-2.1255\times 10^{-16}$
$\beta_{10}$	$4.9736\times 10^{-19}$	$5.0646\times 10^{-19}$	$1.9090\times 10^{-19}$
$\beta_{11}$	$-2.5042\times 10^{-21}$	$4.8947\times 10^{-21}$	$-5.4262\times 10^{-22}$
$\beta_{12}$	$5.7033\times 10^{-24}$	$-2.9292\times 10^{-23}$	$-1.2854\times 10^{-23}$
